# Role of pyroptosis in cardiovascular disease

**DOI:** 10.1111/cpr.12563

**Published:** 2018-12-07

**Authors:** Zeng Zhaolin, Li Guohua, Wu Shiyuan, Wang Zuo

**Affiliations:** ^1^ Yueyang Maternal and Child Health Hospital Yueyang China; ^2^ Institute of Cardiovascular Disease, Key Lab for Arteriosclerology of Hunan Province University of South China Hengyang China

**Keywords:** atherosclerosis, cardiovascular disease, inflammation, pyroptosis

## Abstract

Cardiac function is determined by the dynamic equilibrium of various cell types and the extracellular matrix that composes the heart. Cardiovascular diseases (CVDs), especially atherosclerosis and myocardial infarction, are often accompanied by cell death and acute/chronic inflammatory reactions. Caspase‐dependent pyroptosis is characterized by the activation of pathways leading to the activation of NOD‐like receptors, especially the NLRP3 inflammasome and its downstream effector inflammatory factors interleukin (IL)‐1β and IL‐18. Many studies in the past decade have investigated the role of pyroptosis in CVDs. The findings of these studies have led to the development of therapeutic approaches based on the regulation of pyroptosis, and some of these approaches are in clinical trials. This review summarizes the molecular mechanisms, regulation and cellular effects of pyroptosis briefly and then discusses the current pyroptosis studies in CVD research.

## 1 INTRODUCTION

Cell death (CD) is critical to maintaining tissue homeostasis and basic biological functions, and its changes have significant implications in disease pathology. Since CD was first described in the1960s, many types of CD have been defined based on differences in morphological and biochemical characteristics.^1^ In the past, CD in the cardiovascular system was considered passive and negative. In addition, CD was previously believed to occur due to the loss of function of living cells and subsequent inflammation. This situation has changed with the description of apoptosis in the 1970s. In general, apoptosis is essential to maintain cardiovascular homeostasis. Both reduced and increased apoptosis can result in pathology.^2^

The maintenance of the normal structure and function of the cardiovascular system requires a balance between cell formation and death in the tissues and organs of the cardiovascular system (including cardiomyocytes [CMs], endothelial cells [ECs], vascular smooth muscle cells [VSMCs] and cardiac fibroblasts [CFs]). Excessive CD (including pyroptosis) often leads to dysfunction of tissues and organs.^3^

Pyroptosis was first identified in the macrophage in 1992, which presented rapid lysis after infection with *Shigella flexneri*,^4^ and the name was coined in 2001.^5^ Pyroptosis plays a pivotal role in the pathogenesis of various CVDs and involves ECs,^6^ VSMCs^7^ and so on. This process occurs in patients suffering from myocardial infarction (MI),^8^, ^9^ hypertension^10^, ^11^ and cardiomyopathy,^12^ as well as in animal models of ischaemia–reperfusion injury (IRI),^13^ atherosclerosis (As),^6^ heart failure (HF)^14^ and cardiomyopathy.^15^, ^16^ Pyroptosis is a highly regulated cell death process, and inhibition of this process by pharmacological or genetic intervention is cardioprotective under many conditions.^6^, ^17^ Therefore, this process is a potential target for therapeutic intervention to prevent CVDs. In summary, the discovery of pyroptosis has broadened our understanding of CD in CVDs, and targeting this manner of CD provides new avenues for the treatment and management of CVDs. This review provides a current overview of the evidence and functional role of pyroptosis in CVDs and discusses the molecular pathways involved in the cardiovascular system.

## 2 OVERVIEW OF PYROPTOSIS

Pyroptosis is a form of programmed cell death (PCD), accompanied by an inflammatory response.^18^ PCD refers to the autonomous, ordered death of cells controlled by genes to maintain homeostasis. By contrast, non‐PCD (NPCD) mainly refers to cell necrosis, which involves the passive death of cells upon exposure to physical or chemical stimuli in the environment.^19^ PCD can be blocked by inhibitors of cellular signal transduction, whereas NPCD cannot.^20^ Pyroptosis is triggered by various pathological stimuli, such as oxidative stress, hyperglycaemia (HG), inflammation, and is crucial for controlling microbial infections. At present, pyroptosis can be observed in monocytes, macrophages, dendritic cells, VSMCs, vascular endothelial cells (VECs), CMs, CFs and many other cell types.^21^

Pyroptosis is distinct from other forms of CD, such as apoptosis and autophagy, in morphology and mechanism (Table 1). The main difference between apoptosis and pyroptosis lies in the caspase involved. Apoptotic caspases mainly include caspase‐2,8,9,10 (apoptosis initiation) and caspase‐3,6,7 (apoptosis execution).^22^ Apoptosis does not form a cell membrane pore mediated by Gasdermin D‐N (GSDMD‐N) and releases inflammatory factors.^23^ Kerr et al^24^ first proposed apoptosis in 1972 to describe the disappearance of cells during embryonic development, the morphological pattern of normal adult cell renewal in healthy adult tissues and cell atrophy death after hormone cessation. Apoptosis is a highly regulatable pathway of PCD.^25^ Cysteine protease causes cell matrix lysis, nucleus condensation, DNA cleavage and plasma membrane shrinkage, simultaneously extensive plasma membrane bubbles and numerous apoptosis body are generating. Neither the cytoplasmic contents will be released outside the cell nor any inflammatory reactions triggered during this process.^26^

**Table 1:** Comparison of different forms of cell death and their biological characteristics

	Apoptosis	Pyroptosis	Necroptosis	Necrosis	Autophagy
Triggered by	DR activation (extrinsic)/intracellular signals (intrinsic)	Activation of inflammasomes by pathogens	DR receptor activation upon caspase‐8 inhibition	—	Ulk1‐FIP200‐ATG13 complex Activation
Inflammatory	No	Yes	Yes	Yes	Partially have
Key players	Caspase family (caspase‐8, caspase‐9), Bcl2 family	Caspase‐1, caspase‐4/5/11, Gasdermin D	RIPK1, RIPK3, MLKL	—	ATGs
Death executor	Caspase‐3, caspase‐7	IL‐1β/IL‐18 release	?	—	Autolysosomes
Morphology	Cell rounding, blebbing, formation of apoptotic bodies	Plasma membrane rupture, release of cell contents, maintained mitochondrial integrity	Cell swelling, plasma membrane rupture, release of cell contents	Cell swelling, Plasma membrane rupture, release of cell contents	—
Identification method	Electron microscopy, Flow cytometry, Annexin assay, TUNEL assay, WB, IP, IF	Electron microscopy, Flow cytometry, TUNEL assay, LDH release assay, WB, IP, IF, Hoechst 33342/PI double‐staining	Electron microscopy, flow cytometry, Annexin assay, TUNEL assay, WB, IP, IF	Electron microscopy, LDH release assay, WB, IF, PS exposure/viability dyes	Electron microscopy, WB, IF, flow cytometry, mRFP/mCherry‐GFP‐LC3 fusion protein

ATGs, autophagy‐related genes; DR, death receptor; IF, immunofluorescence; IP, immunoprecipitation; LDH, lactate dehydrogenase; MLKL: mixed lineage kinase domain‐like; PS: phosphatidylserine; RIPK: receptor interacting protein kinase; WB: Western blot.

Autophagy is a conserved intracellular degradation pathway that delivers cytosolic contents to lysosomes via double‐membrane autophagosomes to degrade longevity proteins, misfolded proteins and excess or defective organelles.^27^ Autophagy in most tissues occurs at the basal level and contributes to the renewal of cytoplasmic components. It plays a role in the development, differentiation and tissue remodelling of various organisms. Autophagy is highly induced in pathological conditions and increased by more than 10 times. Cell necrosis is a passive process of acute CD mainly caused by physical and chemical stimulation, often leading to cell swelling, rupture and uncontrolled release of inflammatory contents.^28^

Necroptosis is also a pro‐inflammatory CD that occurs under caspase‐8 inhibition, allowing the activation of the receptor interacting protein kinase 1‐receptor interacting protein kinase 3‐mixed lineage kinase domain‐like axis.^29^ Necroptosis is a regulated form of cell death morphologically characterized by cell and organelle swelling, which ultimately culminates in the loss of plasma membrane integrity and mild chromatin condensation but intact nuclei.^30^

Pyroptosis is characterized by rapid plasma membrane disruption, followed by release of cellular contents and pro‐inflammatory mediators, including IL‐1β and IL‐18.^31^ Unlike most cytokines, IL‐1β and IL‐18 are not secreted by the classical endoplasmic reticulum‐Golgi pathway but are produced as biologically inactive precursor proteins that are cleaved prior to their secretion as bioactive cytokines.^32^ IL‐1β is synthesized initially as an inactive precursor molecule (pro–IL‐1β p35), which must be cleaved by caspase‐1 at amino acid position 116 to produce the actively mature IL‐1β (p17).^33^ Mature IL‐1β is a pro‐inflammatory mediator that recruits innate immune cells to infection sites and modulates adaptive immune cells (T helper 1 [Th1], Th17). Meanwhile, mature IL‐18 is important for the production of interferon‐γ and potentiation of cytolytic activity of natural killer cells and T cells (Th17 cells) and may polarize T cells towards Th1 or Th2 profiles in combination with other cytokines.^34^

**Figure 1:**
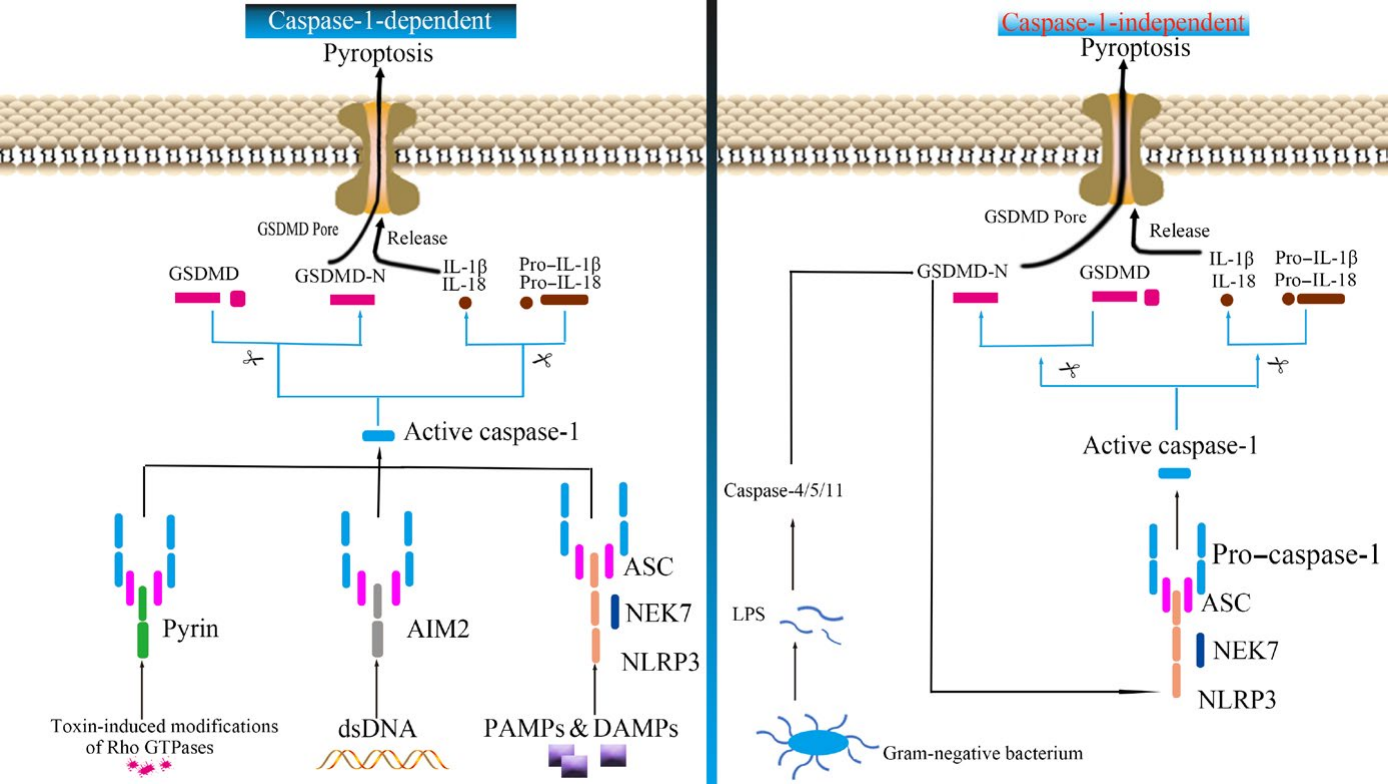
Caspase‐1–dependent and independent pyroptotic pathway. In caspase‐1–dependent pyroptosis pathway, the cells activate their respective inflammasome (including NLRP3, AIM2 or pyrin) through the action of pathogen‐associated molecular patterns (PAMPs) and danger‐associated molecular patterns (DAMPs), under the stimulation of hyperlipidaemia, hyperglycaemia and inflammation; NLRP3 oligomerizes and recruits ASC and pro–caspase‐1, triggering the activation of caspase‐1 and the maturation and secretion of pro‐inflammatory cytokines such as IL‐1β and IL‐18. GSDMD‐N formed by inflammatory caspase cleavage then mediates cell membrane pore formation, and promotes inflammatory factor release, cell swelling and pyroptosis. In caspase‐1–independent pyroptosis pathway, Gram‐negative bacterial cell wall component LPS activates caspase‐4/5/11 pathway to mediate cell pyroptosis

**Figure 2:**
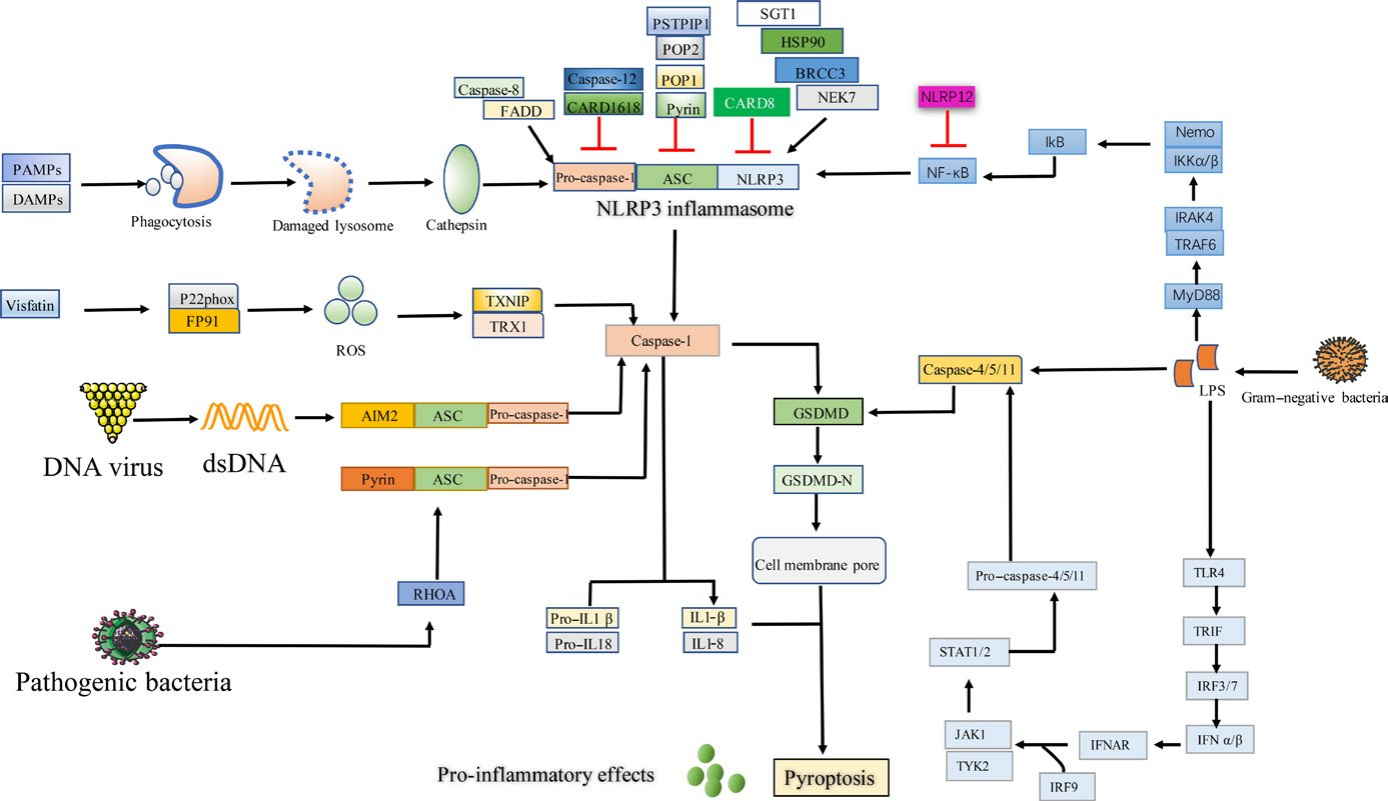
Multiple pathways that mediate pyroptosis. Various factors can activate the inflammasome to trigger pyroptosis. (1) Non‐pathogen stimulating factors such as hyperlipidaemia/hyperglycaemia activate the NLRP3 inflammasome through danger‐associated molecular patterns (DAMPs). NLRP3 interacts with ASC through an N‐terminal PYD domain, which then recruits pro–caspase‐1, promoting the maturation and release of IL‐1β and IL‐18. (2) Pathogen‐stimulated activation of PRRs, such TLR4, activates NF‐κB, leading to the transcription and translation of NLRP3 and then triggers pyroptosis. (3) The AIM2 inflammasome can directly bind double‐stranded DNA (dsDNA) via its HIN200 domain. Binding of dsDNA, leads to AIM2 oligomerization and the recruitment of the adaptor ASC via PYD‐PYD interactions. Once activated, the inflammasomes act as platforms to trigger caspase‐1 expression and the subsequent release of cytokines and pyroptosis. (4) Upon activation of the pyrin inflammasome, pyrin responds to disturbances in cytoplasmic homeostasis caused by infections, and the subsequent inactivation of the RhoA GTPase leads to pyrin activation, inflammasome assembly and pyroptotic cell death. (5) Oxidative stress‐induced phagocytosis of particles or live pathogens leads to lysosome rupture, releasing cathepsin B (CSTB), which facilitates the interaction between NLRP3 and ASC, thereby inducing pyroptosis. (6) The adipokine visfatin activates the NLRP3 inflammasome to trigger inflammasome activation directly or indirectly through an uncharacterized pathway, especially in obesity‐related diseases

Pyroptosis mainly includes the canonical pathway of caspase‐1 dependence and the non‐canonical pathway involving caspase‐4,5 (human) and caspase‐11 (mouse; Figure 1).^35^ The cells activate their respective inflammasomes, including NLRP3, absent in melanoma 2 (AIM2), or pyrin through the action of pathogen‐associated molecular patterns (PAMPs) and danger‐associated molecular patterns (DAMPs) under the stimulation of hyperlipidaemia, HG and inflammation (Figure 2). After the activation of NLRP3, the N‐terminal pyrin domain (PYD) of NLRP3 serves as a scaffold to nucleate apoptosis‐associated speck‐like protein containing a caspase activation and recruitment domain (ASC), which contains a pyrin domain and a caspase activation and recruitment domain (CARD). Through its pyrin domain, ASC interacts with sensor molecules, and the CARD domain interacts with pro–caspase‐1 (p45) and initiates pro–caspase‐1 self‐cleavage to form a caspase‐1 mature body (p10/p20 tetramer). On the one hand, activated caspase‐1 recognizes inactive IL‐β and IL‐18 precursors and converts them into mature inflammatory cytokines. On the other hand, caspase‐1 cleaves GSDMD (a member of the Gasdermin protein family consisting of more than 500 amino acids) and oligomerizes 31 kDa amino‐terminal products (GSDMD‐N) that mediate the formation of membrane pores. The formation of membrane pores promotes the release of inflammatory factors, cell swelling and, finally, pyroptosis.^36^, ^37^

In the non‐canonical pathway (caspase‐1–independent pathway), the Gram‐negative bacterial cell wall component lipopolysaccharide (LPS) is recognized by caspase‐11 in mouse cells and caspase‐4 and caspase‐5 in human cells, and then, caspase‐4/5/11 directly cleaves GSDMD and initiates pyroptosis; meanwhile, the amino‐terminal GSDMD‐N activates NLRP3 inflammasome, but only caspase‐1 processes IL‐1β and IL‐18.^37^, ^38^

Under pathogen stimulation, ligand (like LPS) binding recruits the myeloid differentiation protein 88 (MyD88) adaptor protein to the TIR domain within the cytoplasmic region of IL‐1 receptor I (IL‐1RI) and IL‐18 receptor (IL‐18R), resulting in the recruitment, activation and autophosphorylation of IL‐1R–associated kinase (IRAK). IRAKs are then released from the receptor‐MyD88 complex and couple to the E3 ubiquitin ligase tumour necrosis factor (TNF) receptor‐associated factor 6 (TRAF6), which autoubiquitinates itself and activates TGFβ‐activated kinase 1 (TAK1). TAK1 then activates the IκB kinase complex to release NF‐κB from IκBα‐mediated inhibition and then activates NLRP3 inflammasome to initiate pyroptosis.^15^, ^40^

Nek7 is an essential component of NLRP3 inflammasome activation. Nek7 has been primarily characterized as a factor regulating microtubule network nucleation and spindle formation during mitosis.^41^ In the pyroptosis system, Nek7 could be involved in the formation or provision of a common signal that functions upstream of NLRP3. Nek7 as a regulator of microtubule dynamics could facilitate the interaction between NLRP3 and ASC.^42^

Morphologically, pyroptosis appears to be a combination of apoptosis and necrosis and involves the loss of plasma membrane integrity and the release of cellular contents. The GSDMD‐N domain mediates the formation of small pores with a diameter of about 10‐14 nm in the plasma membrane. Of note, these pores are wide enough for the passage of mature IL‐1β (4.5 nm) and caspase‐1 (7.5 nm). The cells appear to be osmotically swollen and form spherical vesicles around the nucleus.^43^ As the cell expands, the nucleus becomes spherical and condensed, and the DNA fragments. As with apoptosis, the pyroptosis TUNEL assay is also positive. Pyroptosis can also be observed by electron microscopy, lactate dehydrogenase (LDH) release and Hoechst 33342/PI double‐staining, etc.^44^

## 3 INFLAMMASOME ACTIVATION OF INITIAL PYROPTOSIS

### 3.1 NLRP3 inflammasome

The primary function of the innate immune system is to maintain homeostasis, which is partly achieved through immunological surveillance by germline‐encoded pattern recognition receptors (PRRs), such as the Toll‐like receptors (TLRs) and the nucleotide‐binding domain leucine‐rich repeat‐containing receptors (NLRs).^45^ PRRs can be subdivided into two major classes based on their subcellular localization. TLRs and C‐type lectin receptors are transmembrane proteins found in the plasma membrane and endosomes, where they can survey PAMPs and DAMPs in the extracellular milieu. The second class of PRRs resides in intracellular compartments and includes the RIG‐I–like receptor (RLR), the AIM2‐like receptor (ALR) and the NLR proteins.^46^

Inflammasomes are a group of intracellular protein complexes which include NLRs, ASC, caspase‐1 and sometimes caspase‐11.^47^ NLRP family members, including NLRP1, NLRP3, NLRC4, AIM2 and the adaptor ASC, are the critical components of the inflammasome, linking microbial and endogenous “danger” signals to caspase‐1 activation.^37^, ^48^ The best‐characterized inflammasome is the NLRP3 inflammasome multi‐protein complex. It comprises the NLR protein NLRP3, the adapter ASC and the pro–caspase‐1. Once activated, the inflammasomes act as platforms to trigger caspase‐1, cytokine release and pyroptosis.^49^

The mechanism underlying the activation of the NLRP3 inflammasome remains controversial. At present, at least three models are widely accepted. The first model assumes that pore formation allows extracellular NLRP3 agonists to enter the cytosol and directly activate NLRP3. The second mode is mediated by lysosomal rupture. Phagocytosis of particles (such as silica and asbestos) or live pathogens leads to lysosome rupture, releasing cathepsin B (CSTB) or a protein modified by CTSB.^50^ Oxidative stresses are also associated with CTSB activity; excessive ROS generation triggers CTSB release to activate the NLRP3 inflammasome.^51^ Cathepsins are a class of proteolytic enzymes, which display diversity in terms of their structural and/or functional features. The lysosomal cathepsin family can be divided into three subsets, aspartic cathepsins (D and E), serine cathepsins (A and G) and cysteine cathepsins (B, C, F, H, K, L, O, S, V, X and W). The main function in pyroptosis is CTSB. CTSB regulates several processes, including cytokine exocytosis, protein cleavage inside the lysosome and cell death.^52^, ^53^ CTSB in the cytosol is crucial for NLRP3 inflammasome assembly and, consequently, for IL‐1β and caspase‐1 maturation. Cathepsin inhibition abolishes the interaction between NLRP3 and ASC. Furthermore, CTSB has been described in some settings to directly cleave caspase‐1 and caspase‐11, mediating both canonical and non‐canonical pyroptosis.^55^, ^56^ In the third mode, the NLRP3 agonist (eg, HG, hyperlipidaemia) triggers the production of reactive oxygen species (ROS) and further activates the NLRP3 inflammasome.^58^

In addition to the above three modes, the adipokine visfatin (a major injurious adipokine during obesity) is also thought to activate the NLRP3 inflammasome to induce pyroptosis.^59^ This mode plays an important role in vascular endothelial oxidative stress injury and participates in various obesity‐related diseases, such as diabetes^60^ and coronary atherosclerosis.^61^ Moreover, potassium release is associated with all NLRP3 activators, and low‐potassium medium alone is sufficient to trigger NLRP3 activation.^62^ However, whether NLRP3 directly senses this low level of potassium remains to be determined.

Among the three mechanisms, the third one is the most closely related to CVDs. The source of ROS is currently not clearly understood. In general, the main source of intracellular ROS is mitochondria and mitochondria produce ROS including electron transport chains and non‐electron transfer chains.^63^ The electron transport on the mitochondrial electron transport chain is carried out in a step‐by‐step manner, which dramatically increases the chance of ROS production. Complexes I and III on the electron transport chain are considered the primary sites for ROS production. In addition to the site of ROS generation in the electron transport chain, other ROS production sites, such as cytochrome b reductase and monoamine oxidase, exist in the mitochondria.^64^ In addition to the ROS generation system, the body also has a ROS clearance system, which includes ROS scavenging enzymes and antioxidant systems. Under physiological conditions, ROS generation and clearance in the body are in equilibrium. However, when mitochondrial dysfunction occurs, the expression of complexes on the electron transport chain becomes abnormal. This phenomenon increases the probability of electrons escaping from the electron transport chain, causing abnormal ROS production and imbalanced clearance, resulting in excessive ROS generation that causes oxidative stress damage, further aggravating mitochondrial damage and triggering diseases such as, As, HF and so on.^65^, ^66^

### 3.2 AIM2 inflammasome

AIM2 is an innate immune sensor, which contains two major domains: an N‐terminal PYD domain and a C‐terminal HIN domain. It can detect damaged and aberrant DNA within the cytosolic compartment. AIM2 is also a key sensor of pathogens that detects foreign DNA accumulating in the cytosol during the life cycle of intracellular pathogens, including viruses, bacteria and parasites.^67^ A recent study has found that AIM2 can detect DNA damage directly within the nucleus.^68^ AIM2 activation initiates the assembly of the inflammasome, an innate immune complex that activates inflammatory caspases and triggers pyroptosis.^69^ Microbial DNA and host DNA induce caspase‐1 activation dependent of ASC but independent of NLRP3, TLRs or interferon signalling pathway.^70^

### 3.3 Pyrin inflammasome

Pyrin is a large protein (86 kDa) encoded by the MEFV gene located on human chromosome 16 or the mouse analogue Mefv on murine chromosome 16.^7^ Pyrin is mainly found in immune cells, such as neutrophils, monocytes and dendritic cells, and its expression can be up‐regulated by various cytokines, including IFN‐γ, LPS, TNF‐α, IL‐4 and IL‐10.^71^

Pyrin does not directly recognize molecular patterns (pathogen‐ or host‐derived danger molecules) but responds to infection‐induced disturbances in cytoplasmic homeostasis. Pyrin activation is triggered after RhoA GTPase inactivation. Active pyrin inflammasome recruits and activates caspase‐1. Active caspase‐1 promotes the proteolytic maturation and secretion of IL‐1β and IL‐18. At the same time, the product of caspase‐1 cleavage GSDMD, that is GSDMD‐N, forms permeable pores in the cellular plasma membrane, thereby initiating pyroptosis.^72^, ^73^

## 4 PYROPTOSIS IN CARDIOVASCULAR DISEASE

Adult CMs are post‐mitotic cells with insufficient ability to respond to injury. In general, acute injury often leads to various types of CD, whereas chronic stress mainly leads to hypertrophy and myocardial remodelling. Increasing evidence shows a slow turnover in normal myocardium maintained by stem cells. However, under pathological conditions, death beyond the mitosis of CMs leads to heart dysfunction.^2^ Pyroptosis is involved in various CVDs (eg, As, MI and cardiomyopathy) by mediating CD and inflammation. Intervention in pyroptosis‐related molecules (eg, caspase‐1, NLRP3, GSDMD and ASC) can significantly affect CVD progression and outcomes. Therefore, an in‐depth understanding of the role and molecular mechanisms of pyroptosis in CVDs can provide new potential targets for treatment.

### 4.1 Atherosclerosis

As is a chronic progressive disease characterized by abnormal lipid deposition in the aorta, obstructing blood flow and subsequent plaque rupture that causes coronary heart disease (CHD) and stroke. As remains the primary cause of morbidity and mortality worldwide.^74^ Both innate and adaptive immune responses, which mainly involve monocytes, macrophages, neutrophils, T lymphocytes and B lymphocytes, are essential for the initiation and progression of As.^75^ In summary, As may be considered a chronic inflammatory disease caused by interactions among modified lipoproteins, monocyte‐derived macrophages, T cells and ECs. CD can be observed in As and plays a vital role in the development and progression of As lesions. Pyroptosis is involved in the formation and progression of As by promoting the release of inflammatory factors and is closely related to the stability of the plaque.^44^

The most well known of many inflammasomes is NLRP3, which is thought to bridge the gap between lipid metabolism and inflammation because of cholesterol crystals, and oxidized low‐density lipoprotein (oxLDL) can activate the NLRP3 inflammasome to induce pyroptosis. Despite this, there are also reports that the NLRP3 inflammasome does not play an important role in As because the absence of *NLRP3* does not affect the progression of As lesions and plaque stability in high‐fat diet (HFD)‐fed *ApoE^−/−^* mice (a commonly used animal model of As).^76^

#### 4.1.1 Vascular endothelial cell pyroptosis in atherosclerosis

Vascular endothelial cells are barriers between blood and vascular wall. VEC damage is considered the starting point of As lesions. Endothelial injury is often accompanied by different types of CD, such as autophagy, apoptosis, pyroptosis and necrosis. Vascular wall integrity is destroyed after pyroptosis, causing local lipid deposition, As formation and plaque instability, and even acute coronary occlusion and sudden death.^77^ Caspase‐1 is abundantly expressed in human As plaques; in fact, the caspase‐1 content in vulnerable plaques and ruptured lesions in patients who died of acute coronary events is significantly increased.^78^ Thus, pyroptosis is involved in As formation and plaque hardening.

**Figure 3:**
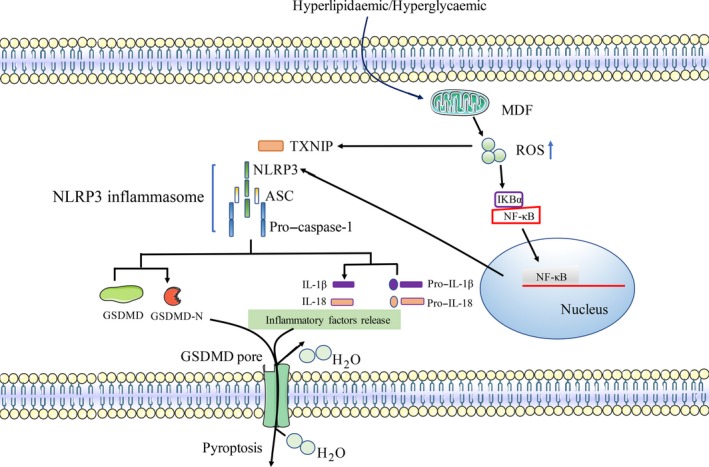
Schematic of the main molecular pathways in pyroptosis in VECs and VSMCs in CVDs. Hyperlipidaemia/hyperglycaemia causes mitochondrial dysfunction (MDF)‐induced generation of reactive oxygen species (ROS), which leads to NF‐κB activation and thioredoxin‐interacting protein (TXNIP) overexpression. NF‐kB–activated NLRP3 inflammasome and maturation of IL‐18 and IL‐1β facilitate inflammatory reaction. Activated caspase‐1 cleaves GSDMD and forms the GSDMD‐N domain, which oligomerizes to generate membrane pores, which disrupt the osmotic potential and lead to cell swelling and eventual lysis

The caspase‐1 inflammasome pathway can sense elevated lipids or inflammatory mediators, such as DAMPs, and then up‐regulate pyroptosis‐related proteins, including NLRP3, caspase‐1 and IL‐1β, eventually triggering VEC pyroptosis. VEC pyroptosis leads to the loss of endothelium integrity, increases vascular permeability and promotes As development. Hyperlipidaemia induces ROS production through an NADPH oxidase‐dependent pathway that activates NLRP3 and caspase‐1, causing VEC pyroptosis and inflammation^6^, ^79^, ^80^ (Figure 3). Early hyperlipidaemia promotes monocyte recruitment and EC activation through the caspase‐1–sirtuin 1‐activator protein‐1 pathway and exacerbates As. In the *caspase‐1^−/−^* and* ApoE^−/−^*double knockout mice, the recruitment of monocyte is inhibited and reduces the expression of adhesion molecules and the secretion of cytokines and inflammatory cytokines.^81^, ^82^ This result provides new insights for target drug development in As.

Cigarette smoking is a major risk factor for As and other CVDs, but its underlying mechanism remains unclear. Wu et al^79^ found that nicotine promotes As by inducing VECs pyroptosis. Nicotine can increase ROS generation, activate NLRP3 inflammasome, cleave pro–caspase‐1 and induce the production of downstream IL‐1β and IL‐18, which are all inhibited by caspase‐1 inhibitor. In the *ApoE^−/−^* mouse model, nicotine exposure promotes As development by increasing VEC pyroptosis. Silencing *NLRP3* expression can reduce As lesion area and lipid deposition in the aortic root.

Recent studies support the role of mitochondrial adaptors, mitochondrial calcium fluxes and mitochondrial ROS (mtROS) generation in inflammasome activation.^83^ Mitochondrial‐derived mtROS is a major source of cellular ROS, and excessive mtROS is associated with As progression in both human and mouse models.^64^ Cadmium is a relatively common environmental metal pollutant that can cause As and hypertension. Cadmium activates NLRP3 inflammasome and downstream caspase‐1 and IL‐1β production by inducing mtROS, which mediates the pyroptosis of VECs and promotes the development of As.^84^

MicroRNAs (miR) are endogenous, small non‐coding RNAs of approximately 22 nucleotides in length that anneal inexactly to complementary sequences in the 3′‐untranslated regions (3′‐UTR) of target mRNAs to either facilitate their degradation or repress their translation.^85^ In the present study, we find that miR‐125a‐5p mediates oxLDL‐induced pyroptosis in VECs by down‐regulating tet methylcytosine dioxygenase 2 (TET2), increasing NF‐κB activation, activating NLRP3 and caspase‐1 p20, and ultimately causing pyroptosis of VECs. After TET2 down‐regulation, abnormal DNA methylation occurs, and mitochondrial dysfunction subsequently induces ROS production, which activates the NLRP3 inflammasome, leading to the activation of caspase‐1. Activated caspase‐1 promotes GSDMD oligomerization, which triggers pore formation of the membrane, DNA fragmentation and release of mature IL‐1β and IL‐18 from cells, causing a sterile inflammatory response and further contributing to pyroptotic cell death and subsequently promoting As.^86^

#### 4.1.2 Monocyte/macrophage pyroptosis in atherosclerosis

The role of innate and adaptive immune factors in As is gradually being valued. As plaques are characterized by lipid deposition in the arterial wall, infiltration of immune cells (eg, macrophages, T cells and mast cells) and fibre cap (mainly composed of smooth muscle cells, collagen fibres, elastic fibres and proteoglycans). In early lesions called “fatty streaks,” lipid deposition and macrophage foaming can be observed, and complex lesions appear with time, accompanied by apoptosis and necrosis. The necrotic core is covered by a fibrous cap, and its “shoulder” region is infiltrated by activated T cells, macrophages and mast cells, which produce pro‐inflammatory mediators that can render plaques unstable and can cause rupture of the fibrous cap, leading to vascular embolization and tissue infarction.^87^ Although the death of macrophages in early As lesions is beneficial, the reduction in the number of these cells in the plaque can attenuate the inflammatory response and reduce the synthesis of matrix metalloproteinases. However, death of macrophages in advanced lesions promotes the formation of necrotic cores and the instability of As plaques. Macrophage death in As lesions causes the release of growth factors, cytokines, proteases and intracellular lipids to the inflammatory response; promotes plaque rupture and thrombosis; and causes acute cardiovascular events.^88^, ^89^

**Figure 4:**
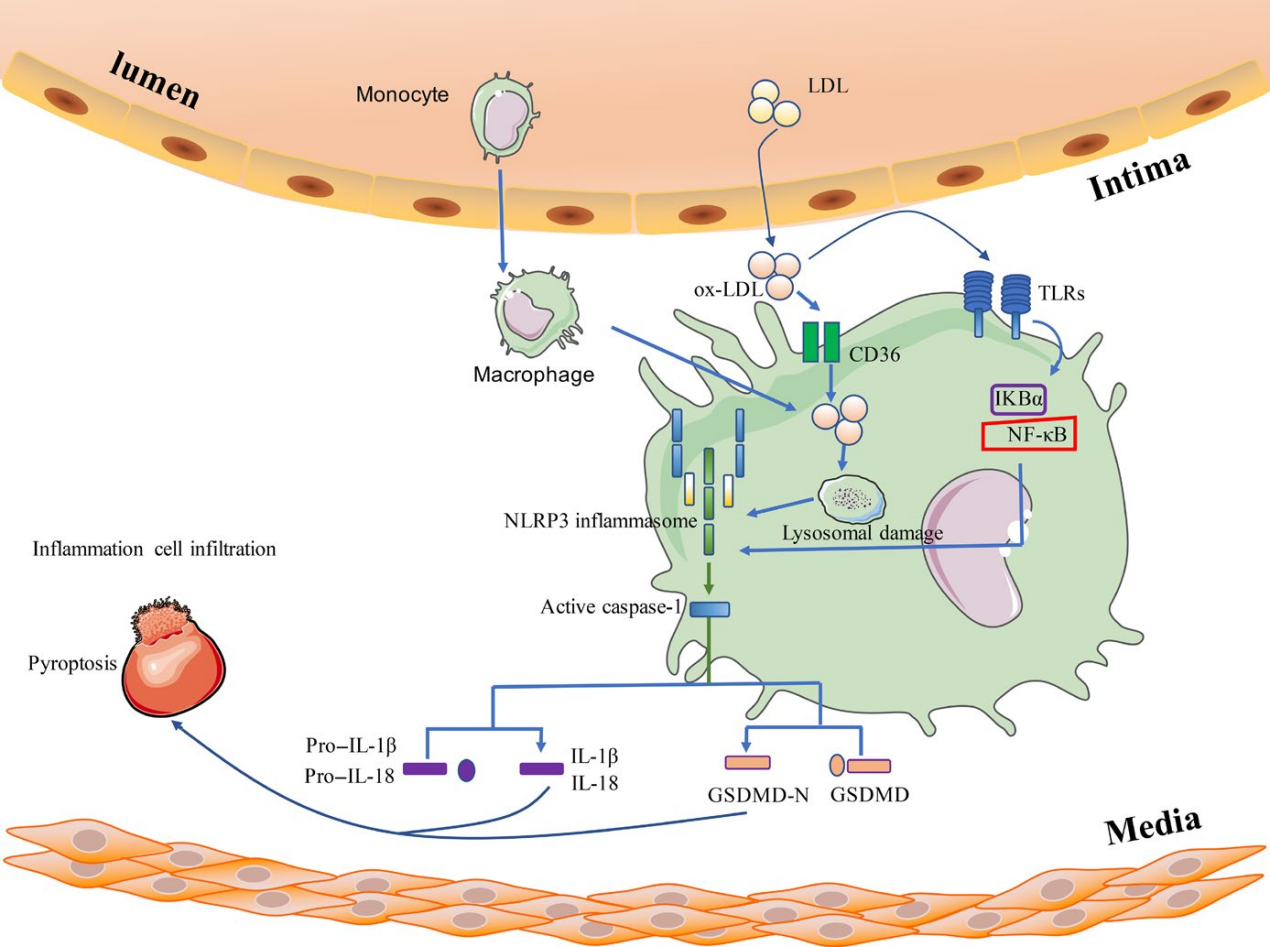
Schematic of the primary molecular pathways leading to pyroptosis in monocytes/macrophages in CVDs. Internalization of oxidized low‐density lipoprotein (oxLDL) by macrophages through CD36 receptor leads to intracellular gathering of cholesterol crystals in large phagolysosomal compartments. Cholesterol crystals can activate the NLRP3 inflammasome through lysosomal damage. oxLDL can also activate the NLRP3 inflammasome via the TLRs/NF‐κB pathway. Activated NLRP3 inflammasome induces the release of mature IL‐1β and IL‐18 and further leads to inflammatory cell infiltration and pyroptosis

Serum total cholesterol and low‐density lipoprotein cholesterol (LDL‐C) are risk factors for CHD, and oxLDL has a stronger effect to As.^90^ OxLDL‐induced macrophage pyroptosis plays an important role in As formation and plaque stability. OxLDL and cholesterol crystals in the plaque necrosis area can activate NLRP3 and caspase‐1 to induce cell pyroptosis. This phenomenon causes the release of IL‐18 and IL‐1β in mouse macrophages, which exacerbates inflammation and As^91^ (Figure 4). Triglycerides are also another As risk factor, which can trigger pyroptosis and aggravate the disease.^92^

IL‐1β of the IL‐1 family is an important pro‐inflammatory cytokine that is mainly produced by activated monocytes/macrophages.^93^ On the one hand, IL‐1β activates monocytes, macrophages and neutrophils; on the other hand, it induces Th1 and Th17, thereby participating in the initiation of inflammatory and immune responses.^81^ Depletion of *IL‐1β* inhibits As and reduces the area of As plaque in *Apo E^−/−^* and *IL‐1^−/−^* mice by approximately 30% compared with the control group of *Apo E^−/−^* mice.^94^ The lack of mitochondrial DNA (mtDNA) can also inhibit the formation and development of As. RHO0 cells (a type of cell line without mtDNA) can resist apoptosis, and such mtDNA‐depleted cells can resist oxLDL‐induced cell pyroptosis possibly by reducing the production of ROS and then inhibiting the activation of NLRP3 inflammasomes. Current studies have also shown that absence of mtDNA does not affect oxLDL‐induced intracellular lipid accumulation and mitochondrial membrane potential.^95^ Mitochondria are closely related to CD, and the disruption of mitochondrial transmembrane potential (Δψ) is considered an early indication of the apoptotic cascade. This disruption occurs before the appearance of apoptotic features in the nucleus (chromatin condensation and DNA fragmentation). Once the mitochondrial transmembrane potential collapses, apoptosis becomes irreversible.^96^ However, the exact mechanism of the anti‐pyroptotic effects of RHO0 cells remains unclear.

CD36 is a membrane glycoprotein present on various cell types, including monocytes, macrophages, microvascular ECs, adipocytes and platelets. Macrophage CD36 participates in the formation of As lesions by interacting with oxLDL. CD36 can play a role in oxLDL uptake and foam cell formation, which is the initial critical phase of As. In vitro and in vivo experimental studies have shown that deletion of *CD36* can inhibit the formation of As lesions. Platelet CD36 also promotes the inflammation of As and participates in the formation of thrombus after the rupture of As plaques. Sheedy et al^97^ found that deletion of *CD36* suppresses the production of IL‐1β, and targeted inhibition of CD36 can reduce the plasma concentration of IL‐1β and the deposition of cholesterol crystals in As plaque, thereby inhibiting As progress. On the basis of the above reasons, studying the function of CD36 and its corresponding signalling pathway may become a new strategy for the treatment of As.

A strong epidemiologic correlation exists between periodontal disease and CVDs, but the exact mechanism remains unclear. Human periodontal disease pathogen *Porphyromonas gingivalis* (Pg) interacts with innate immune receptor Toll‐like receptor 2 (TLR2) and CD36/scavenger receptor‐B2 (SR‐B2). CD36/SR‐B2 and TLR2 promote NLRP3 inflammasome activation and IL‐1β production, thereby inducing pyroptosis and promoting the formation and development of As.^98^

Sirtuin 1 (SIRT1), an NAD^+^‐dependent deacetylase, is a key regulator in inflammatory response during CVDs.^99^ Cluster of differentiation 40 (CD40), a molecule with pro‐inflammatory properties, is a TNF receptor superfamily member, which participates in inflammatory events of CVDs.^100^ Li et al^101^ found that the level of SIRT1 is reduced in ECs following LPS and ATP treatments. LPS and ATP trigger the activation of the NLRP3 inflammasome. Activation of SIRT1 inhibits NLRP3 inflammasome activation and subsequent pro–caspase‐1 cleavage and IL‐1β secretion by inhibiting the expression of CD40.

#### 4.1.3 Pyroptosis of vascular smooth muscle cells in atherosclerosis

Vascular smooth muscle cells participate in the repair of vascular injury through phenotypic and functional transformation. Activated VSMCs have enhanced proliferation and migration abilities, which contribute to the repair of blood vessel walls. However, in chronic inflammation of As, the phenotype and function of arterial VSMCs become abnormal, which leads to VSMC differentiation and increased extracellular matrix formation in the plaque region.^102^

AIM2, a member of the HIN‐200 protein family, plays a crucial role in activating inflammasomes. Pan et al^7^ reported that HFD increases the expression of AIM2, GSDMD‐N and intercellular cell adhesion molecule‐1 (ICMA‐1). *AIM2* overexpression increases the plaque lesion area and VSMC pyroptosis, thereby aggravating As. Moreover, macrophage recruitment is increased with overexpression of *AIM2*. In vitro studies showed that AIM2 expression is associated with NF‐κB signalling activity. AIM2 mediates GSDMD activity through the ASC, caspase‐1 pathway.

Vascular smooth muscle cells pyroptosis is closely related to the stability of As plaques. Molecules involved in cell pyroptosis (NLRP3, ASC, caspase‐1, IL‐1β and IL‐18) are more expressed in unstable plaques than in stable ones.^103^ Bauriedel et al^104^ found more apoptotic VSMCs in unstable than in stable As plaques. In the arterial intima, the extracellular matrix produced by VSMCs, including collagen and elastin, is an important component of the fibrous cap, which constitutes As plaque. This component is closely related to plaque stability and acute cardiovascular events. A large number of VSMCs and macrophages die in the late lesions of As. The death of VSMCs in As can render the fibrous cap of the plaque fragile and unstable and induce acute coronary syndrome.^82^

### 4.2 Ischaemic heart disease

Many CVDs accompany CD, and As and MI may be the most closely related to pyroptosis because they are often accompanied by CD and inflammation. Inhibition of the NLRP3 inflammasome or ASC can reduce infarct size and improve cardiac function in an animal model of MI.^7^, ^105^

MI is the leading cause of death worldwide. Rapid diagnosis and reperfusion significantly improve the survival rate of MI patients. However, cardiac IRI after reperfusion therapy (thrombolysis, percutaneous coronary intervention, coronary artery bypass grafting) triggers significant tissue destructive and aseptic inflammatory responses. Increased risk of HF, myocardial stunning, arrhythmia, myocardial microvascular dysfunction and death limits the beneficial effects of reperfusion. Therefore, preventing and alleviating IRI are expected to improve the prognosis of patients with acute MI.^106^, ^107^

A recent study has revealed an important endogenous inhibitor of inflammation, namely activated protein C (aPC), which can reduce infarct size in mice with MI. In vitro, aPC inhibits NLRP3 inflammasome activation in macrophages, CMs and CFs via proteinase‐activated receptor 1 (PAR‐1) and mammalian target of rapamycin complex 1 signalling. The mTOR pathway is related to energy metabolism, and mTOR activation can inhibit autophagy.^108^ PARs are members of the G protein–coupled family, and four members of the PAR family have been discovered so far: PAR1‐4. PARs represent a component of the innate inflammatory response, being involved in neutrophil recruitment, increased perfusion, pain and swelling. They reportedly serve as the first “alert system” for bacterial invasion. PAR1 is expressed by platelets, osteoblast, ECs, epithelial cells, fibroblasts, myocytes, neurons and astrocytes, and it plays an important role in injured tissues.^109^

Diabetes is one of the risk factors for CVDs. Accompanied by mitochondrial swelling and sarcoplasmic reticulum expansion, left ventricular ultrastructure abnormalities and myocardial fibrosis are more severe in diabetic MI rats compared with non‐diabetic rats. The creatine kinase isozyme CK‐MB and LDH release are significantly higher in diabetic rats than in non‐diabetic MI rats under the same conditions. HG promotes NLRP3 inflammasome‐mediated pyroptosis and aggravates IRI by causing mitochondrial dysfunction, leading to enhanced ROS production. ROS induces the release of inflammation‐related signalling factors, such as NF‐κB, and the subsequent NLRP3 inflammasome activation triggers sterile inflammation and pyroptosis.^110^ Activation of inflammasomes can be inhibited by antioxidants, such as SIRT‐1. Inhibition of the NLRP3 inflammasome or reduction of ROS production can significantly reduce myocardial IRI.^13^, ^111^

In addition to CMs, CFs also play an important role in the maintenance of cardiac physiological functions. CFs are the most abundant cell type in the adult human heart, and they considerably affect the structure and function of the heart.^112^ Øystein et al reported that the NLRP3 inflammasome is up‐regulated in CFs and mediates myocardial IRI.^113^ However, the role of CFs in CVDs remains to be further studied.

### 4.3 Diabetic cardiomyopathy

Diabetic cardiomyopathy (DCM) is one of the major complications of diabetes and is also the leading cause of death in diabetic patients. DCM is characterized by structural and functional impairments, including cardiomyocyte death, cardiac fibroblast activation, left ventricular dysfunction and metabolic disorders. Among them, the death of CMs and CFs is considered a fundamental change in DCM, which initiates cardiac remodelling and leads to left ventricular dysfunction.^114^

#### 4.3.1 Cardiomyocyte pyroptosis in DCM

Hyperglycaemia‐induced ROS overproduction promotes the activation of the NLRP3 inflammasome by NF‐kB and thioredoxin‐interacting protein (TXNIP).^16^ TXNIP, also known as thioredoxin binding protein‐2, is a ubiquitously expressed protein that interacts and negatively regulates the expression and function of thioredoxin (TXN). TXNIP is closely related to energy metabolism. TXNIP influences glucose metabolism by affecting hepatic glucose production and peripheral glucose uptake and regulating beta cell function. In addition, overexpression of TXNIP induces the apoptosis of pancreatic β‐cells, reduces the sensitivity of peripheral tissues, such as skeletal muscle and fat, to insulin, and minimizes energy expenditure.^15^, ^115^

The inflammatory response is involved in the development of DCM. Studies have shown that IL‐1β is an essential pro‐inflammatory cytokine in the development of DCM. The NLRP3 inflammasome also plays a crucial role in the inflammatory process in diabetic nephropathy and retinopathy.^116^

In mammals, the Hu/ELAV RNA binding protein family consists of four highly conserved members, including HuR/HuA/Elavl1 and the neuronal‐specific Hel‐N1/HuB/Elavl2, HuC/Elavl3 and HuD/Elavl4. All family members contain an RNA recognition motif with high affinity for U‐ and AU‐rich sequences (AREs).^117^ ELAVL1 is a member of the RNA binding protein family, which binds to ARE‐ and U‐rich element (URE)‐containing sequences and stabilizes mRNAs. ELAVL1 plays a critical role in the progression of inflammation and HF. In HG state, ELAVL1 expression is increased in ventricular myocytes, which mediates TNF‐α–induced myocardial pyroptosis. Furthermore, ELAVL1 is the target gene of miR‐9, and miR‐9 expression is significantly down‐regulated in high glucose (HG)–treated CMs and human diabetic hearts. ELAVL1 knockdown or miR‐9 mimic transfection suppression of pyroptosis reduces caspase‐1 and IL‐1β expression.^118^

Li et al^119^ found the miR‐30d expression is significantly increased in streptozotocin‐induced diabetic rats and HG‐treated CMs. miR‐30d promotes cardiomyocyte pyroptosis through direct targeting of forkhead box O3 (Foxo3a), a key regulator of cell cycle arrest, oxidative scavenging, cell proliferation, survival and death. miR‐30d also decreases the expression of apoptosis repressor with caspase recruitment domain (ARC). ARC is a transcriptional target of foxo3a in CMs. ARC reduces apoptosis in rabbit IRI models. By contrast, genetic ablation of *ARC* accelerates cardiomyopathy in the context of IRI.^120^

#### 4.3.2 Pyroptosis of cardiac fibroblasts in DCM

NLRP3 inflammasome components have been identified in both CMs and CFs, which are the two most abundant cell populations in the mammalian heart. The fibroblast can thus be a friend in normal function or a foe in pathophysiological conditions. Given their potential role in the regulation of global myocardial function, CFs represent an attractive therapeutic target in heart disease.^108^ Cardiac fibrosis is one of the predominant pathological features of diabetic cardiomyopathy, and CFs play an important role in this process.^121^

Long non‐coding RNA is a type of RNA with a length of >200 nucleotides and non‐coding protein.^122^ In recent years, many reports have focused on the association of lncRNA with pyroptosis in CVDs, such as maternally expressed gene 3 (MEG3)^6^ and metastasis‐associated lung adenocarcinoma transcript 1 (MALAT1).^17^ The KCNQ1 opposite strand/anti‐sense transcript 1 (Kcnq1ot1) is a lncRNA located in human chromosome 11p15.5.^123^ Kcnq1ot1 is involved in various CVDs, including acute myocardial damage^124^ and arrhythmia.^125^ Silencing *Kcnq1ot1* ameliorates cardiac function and fibrosis in diabetic mice. Kcnq1ot1 and pyroptosis are activated in HG‐treated CFs, and silencing Kcnq1ot1 inhibits pyroptosis. Kcnq1ot1 functions as a competing endogenous RNA to regulate the expression of caspase‐1 by sponging miR‐214‐3p.^126^

Zhang et al^127^ demonstrated that H3 relaxin inhibits HG‐induced collagen synthesis through ROS and P2X7 receptor (P2X7R)‐mediated NLRP3 inflammasome activation in neonatal rat CFs, alleviating cardiac fibrosis in DCM. H3 relaxin is an active peptide, which plays a protective role in CVDs. Exogenous H3 relaxin exerts anti‐fibrotic actions via relaxin family peptide receptors 1 and may enhance the collagen inhibitory effects of H2 relaxin.^128^ P2X7R is expressed on CFs and is activated by extracellular ATP to induce pyroptosis.^129^

### 4.4 Cardiac hypertrophy

The impairment of cardiac function caused by cardiac hypertrophy severely affects the quality of life of patients, but the underlying molecular mechanism remains unclear. In addition to hypertrophic cardiomyopathy, the most common cause of ventricular hypertrophy is long‐term uncontrolled systolic hypertension and heart valve stenosis.^130^ At the cellular level, cardiac hypertrophy usually manifests as an increase in the number of CMs accompanied by cytoskeletal remodelling. At the molecular level, cardiac hypertrophy up‐regulates the expression of foetal genes. Physiologically, cardiac hypertrophy is the initial adaptive response to pressure overload. However, persistent pressure overloads often cause HF and even sudden death, leading to poor patient outcomes.^130^

Caspase‐1 and IL‐1β expression levels are significantly up‐regulated in hypertrophic cardiomyocytes both in vivo and in vitro, and the inhibition of caspase‐1 can mitigate cardiac hypertrophy induced by angiotensin II, which offers a therapeutic potential against cardiac hypertrophy.^93^

## 5 ADVANCES IN DRUG APPLICATION TO IMPROVE CVDS BY INTERFERING PYROPTOSIS

Resveratrol (RSV) is a natural polyphenol, which protects heart tissue from damage and has anti‐inflammatory, antioxidant, anti‐ageing and anti‐cancer properties.^85^ RSV can alleviate DCM by inhibiting the activation of the NLRP3 inflammasome by suppressing TXNIP and the phosphorylation of mitogen‐activated protein kinase signalling pathways. Moreover, silencing *NLRP3* can ameliorate cardiac remodelling and dysfunction.^131^

Sinapic acid (SA), also known as 4‐hydroxy‐3,5‐dimethoxycinnamic acid, is an effective component of the seeds of Chinese traditional herbs sinalbin (*Brassica alba* (L.) Boiss) and yellow mustard (*Brassica juncea* (L.) Czern. et Coss.). Recent studies have found that low‐dose (≤50 mg/kg) SA can inhibit As in diabetic model mice by down‐regulating the expression of lncRNA‐MALAT1, inhibiting the activation of NLRP3 inflammasome and inhibiting the macrophages from pyroptosis. Low doses of SA can also reduce the levels of endothelin‐1 and IL‐1β. In macrophages incubated with HG and oxLDL, they have similar effects on knockdown of MALAT1 and 1 μmol of SA.^17^

Trimetazidine (TMZ) is an anti‐ischaemic drug that significantly reduces intracellular acidosis and apoptosis, thereby protecting mitochondrial function and myocardium; TMZ is widely used to treat MI and other ischaemic heart diseases.^132^ Sepsis is a life‐threatening organ dysfunction syndrome caused by a host’s dysfunctional response to infection and is one of the most common causes of death in hospitalized patients.^133^ Cardiac dysfunction is a common complication of sepsis and an important cause of death.^134^ Chen et al^135^ reported that TMZ attenuates LPS‐induced cardiomyocyte pyroptosis and cardiac dysfunction through promoting neutrophil recruitment to heart tissues by regulating chemokine CXC receptor 2 expression through the AMPK/Nrf2 pathway. This result suggests that TMZ is a potential therapeutic agent for septic cardiac dysfunction.

Melatonin (*N*‐acetyl‐5‐methoxytryptamine), a neuroendocrine hormone mainly synthesized in the pineal, participates in sleep‐wake regulation and involved in many diseases, including CVDs.^136^ Melatonin is a potent antioxidant with anti‐atherogenic effects. Latest research findings showed that administration of melatonin for 12 weeks markedly reduces As plaque and lipid content (by 55%) in HFD‐treated *ApoE^−/−^*mice. Melatonin attenuates the expression of pyroptosis‐related genes, including *NLRP3*, *ASC*, cleaved‐*caspase‐1*, *GSDMD*, *IL‐1β* and *IL‐18*, in aortic endothelium. Melatonin also prevents pyroptosis in oxLDL‐treated human aortic ECs (HAECs). Moreover, lncRNA MEG3 is significantly increased in the endothelium of HFD‐treated *ApoE^−/−^* mice, and MEG3 acts as an endogenous sponge by sequence complementarity to suppress the function of miR‐223 and to increase NLRP3 expression and enhance HAEC pyroptosis. Knockdown of miR‐223 blocks the anti‐pyroptotic actions of melatonin in oxLDL‐treated HAECs.^6^ MEG3 is an imprinted gene, which is located on the human chromosome 14q32.3 in the imprinted DLK1‐MEG3 locus and contains 12 gene isoforms encoding lncRNAs associated with tumorigenesis. In addition, MEG3 is a lncRNA, which regulates angiogenesis and differentiation and is associated with diabetes‐associated microvascular dysfunction. Silencing *MEG3 *may exacerbate retinal vascular dysfunction.^137^

Dihydromyricetin (DHM) is a natural flavonoid with antioxidant and anti‐inflammatory effects. DHM can inhibit the palmitic acid (PA)‐induced pyroptosis of VECs by activating the Nrf2 signalling pathway to inhibit PA‐induced intracellular ROS production; thus, it may also have anti‐As action.^18^

Myocarditis is an inflammation of the myocardium caused by various factors (including viral and microbial infections and non‐infectious factors) that can lead to HF and death.^107^ Cholecalciterol cholesterol emulsion (CCE), a precursor of 1,25(OH)2 D3, is used in clinical practice to cure infant rickets caused by vitamin D deficiency. Vitamin D is a potent inhibitor of the inflammatory response, and calcitriol binds to vitamin D response elements in the ASC promoter region to modulate the transcriptional activity of ASC and down‐regulate the pyroptosis signalling pathway. CCE can improve mitochondrial function, inhibit pyroptosis and ameliorate experimental autoimmune myocarditis.^138^

In isoproterenol (ISO)‐induced cardiac fibrosis mouse model, the mRNA expression levels of NLRP3, caspase‐1 and IL‐18 are significantly down‐regulated by 200 mg/kg astragaloside IV, and its active sapogenin cycloastragenol (62.5 mg/kg), thus, inhibit ISO‐induced cardiac fibrosis.^139^

TNF‐α is a pleiotropic cytokine produced after myocardial ischaemia and plays an important role in triggering inflammation and cardiac systolic dysfunction. The inflammatory response centred on TNF‐α plays a leading role in the pathogenesis of micro coronary embolism. TNF‐α triggers the activation of NF‐κB, which increases the transcription and translation of NLRP3 inflammasome components, and participates in pyroptosis.^140^ The glucagon‐like peptide‐1 (GLP‐1) analogue liraglutide stimulates insulin secretion by binding to the GLP‐1 receptor on islet β cells and inhibits the secretion of glucagon from pancreatic islet α cells.^141^ Liraglutide has strong cardiovascular effects. Liraglutide suppresses TNF‐α and hypoxia‐induced pyroptosis in H9c2 cells by up‐regulating SIRT1 and reduces the expression of the ROS inducer protein NOX4, thereby attenuating ROS generation. This phenomenon inhibits NLRP3 inflammasome activation. Tip liraglutide may play a protective role in myocardial ischaemia.^142^

## 6 CONCLUSION AND PERSPECTIVES

CD under physiological conditions (such as proper autophagy and apoptosis) is necessary to maintain tissue renewal, but CD in pathological conditions (such as excessive or insufficient autophagy, necrosis and pyroptosis) leads to disease occurrence and development. CVDs are diseases with the highest morbidity and mortality in the world.^74^ In the cardiovascular system, the pyroptosis of VECs causes the disintegration of blood vessel walls, which consequently promotes the occurrence of As and embolism induced by lipid deposition.^143^ The pyroptosis of VSMCs results in unstable atherosclerotic plaques, acute coronary syndrome and stroke; monocytes/macrophages pyroptosis aggravates the inflammatory response and promotes the development and progression of various CVDs (such as As and MI).^144^

In the recent decade, research on pyroptosis and CVDs has progressed rapidly. Numerous studies have confirmed the important role of pyroptosis in CVDs, in which various stimuli (such as high‐fat/high‐sugar) lead to mitochondrial dysfunction. In turn, ROS overproduction, increased nuclear translocation of NF‐κB and activated NLRP3 inflammasome inducing pyroptosis occur extensively in ECs, CMs and VSMCs. Interfering with related molecules in the pyroptotic pathway (eg, NLRP3, AIM2, caspase‐1 and IL‐1β) affects the occurrence and progression of pyroptosis and CVDs, which may provide a potential treatment target for CVDs (Table 2). A 4‐year clinical trial on pyroptosis, which involves 100 participants and is expected to end in 2020, aims to study the role of pyroptosis in acute lung injury. (For more details, check out this site https://clinicaltrials.gov/ct2/show/NCT03227107?cond=pyroptosis&rank=1; search for qualifier is “pyroptosis,” visited this page on 23 June 2018.).

**Table 2:** Summary of the different agent effect on pyroptosis in cardiovascular system in vivo and in vitro

Agent	Animal/cell model	Pathway	Promote(+)/Suppress(−) CVDs	References
Nicotine	*ApoE^−/−^*mice/HAECs	ROS‐NLRP3‐ASC	+	79
Melatonin	*ApoE^−/−^*mice/HAECs	MEG3/miR‐223/NLRP3	−	6
TG	THP‐1 macrophages	P38 MAPK/caspase‐1	+	92
oxLDL	HUVECs and HAECs	ROS/NF‐κB/NLRP3	+	6, 79
mtDNA absence	J774A.1 RHO0 cells	ROS/NLRP3	−	95
CD36 absence	*Cd36^−/−^*, *Tlr4^−/−^*, *Tlr6^−/−^*, *Nlrp3^−/−^* and *Ice1^−/−^*, *Apoe^−/−^* and *Cd36^−/−^* *ApoE^−/−^* mice/macrophage	CD36/TLR4‐TLR6/NLRP3	−	97
Overexpress AIM2	*ApoE^−/−^*mice/VSMCs	AIM2/NF‐κB/GSDMD	+	7
Cadmium	HUVECs	mtROS/NLRP3	+	84
Porphyromonas gingivalis	CD36*^−/−^*/Ldlr*^−/−^* Ldlr*^−/−^* mice/Macrophage	TLR2‐CD36/SR‐B2/IL‐1β	+	98
Sinapic acid	Type 2 diabetic rat model/Macrophages	MALAT1/miR‐23c/NLRP3/IL‐1β	−	17
NLRP3 knockout	*ApoE^−/−^* *& NLRP3^−/−^* mice,* ApoE^−/−^ & ASC^−/−^* mic， *ApoE^−/−^ and caspase‐1^−/−^* mice	NLRP3/ASC/caspase‐1	No significant effect	76
HG	Type 2 diabetic rat model/H9c2 cardiomyocytes	ROS/NF‐κB/TXNIP/NLRP3	+	15, 16
Trimetazidine	Sepsis C57/BL6 mice/Co‐culture of BMDNs and CMs	AMPK/Nrf2/CXCR2	−	135
Resveratrol	Type 2 diabetes in rats	NLRP3/MAPK	−	131
H3 relaxin	Type 2 diabetes in Rats/CFs	ROS/P2X7R	−	127

AIM2: absent in melanoma 2; ASC: apoptosis‐associated speck‐like protein containing a caspase activation and recruitment domain; BMDNs: bone marrow‐derived neutrophils; CFs: cardiac fibroblasts; CMs: cardiomyocytes; GSDMD: Gasdermin D; HAECs: human aortic endothelial cells; HG: hyperglycaemia; HUVECs: human umbilical vein endothelial cells; IL: interleukin; MAPK: mitogen‐activated protein kinase; MEG3: Maternally expressed gene 3; NF‐κB: nuclear factor κB; NLRP3: the nucleotide‐binding oligomerization domain‐like receptor family pyrin domain‐containing 3; oxLDL: oxidized low‐density lipoprotein; ROS: reactive oxygen species; TG: triglyceride; TLRs: Toll‐like receptors; VSMCs: vascular smooth muscle cells.

In the present review, in addition to the As, we mainly summarize the role of cardiomyocyte pyroptosis in CVDs. However, non‐myocyte cell types in the myocardium, including CFs and mast cells, also play a crucial role in CVDs (eg, hypertensive heart disease and MI). The heart is composed of 70% non‐myocytes and 30% myocytes.^112^ So far, few studies investigated the role of non‐myocytes pyroptosis in CVDs. Thus, this research gap may be the focus of future studies. In the following years, we expect the development of new approaches on controlling the different forms of CD in clinical practice to provide new treatment strategies for patients with CVDs.

## CONFLICT OF INTEREST

The authors declare that they have no conflict of interest.
